# Analysis of optimal alignments unfolds aligners’ bias in existing variant profiles

**DOI:** 10.1186/s12859-016-1216-1

**Published:** 2016-10-06

**Authors:** Quang Tran, Shanshan Gao, Vinhthuy Phan

**Affiliations:** Department of Computer Science, University of Memphis, Memphis, 38152 TN USA

**Keywords:** Short read alignment, Variant calling, INDEL detection

## Abstract

Efforts such as International HapMap Project and 1000 Genomes Project resulted in a catalog of millions of single nucleotides and insertion/deletion (INDEL) variants of the human population. Viewed as a reference of existing variants, this resource commonly serves as a gold standard for studying and developing methods to detect genetic variants. Our analysis revealed that this reference contained thousands of INDELs that were constructed in a biased manner. This bias occurred at the level of aligning short reads to reference genomes to detect variants. The bias is caused by the existence of many theoretically optimal alignments between the reference genome and reads containing alternative alleles at those INDEL locations. We examined several popular aligners and showed that these aligners could be divided into groups whose alignments yielded INDELs that agreed strongly or disagreed strongly with reported INDELs. This finding suggests that the agreement or disagreement between the aligners’ called INDEL and the reported INDEL is merely a result of the arbitrary selection of one of the optimal alignments. The existence of bias in INDEL calling might have a serious influence in downstream analyses. As such, our finding suggests that this phenomenon should be further addressed.

## Introduction

The International HapMap Project and 1000 Genomes Project [1, 2] produced over 10 million single nucleotide variations (SNV) and approximately one million insertion/deletion (INDEL) of the human population. This resource has been utilized to develop a nearly complete map of haplotypes of the human genome [3] and to discover the great extent to which diseases are affected by human genetics. Various approaches for detecting variants have been developed [4–12]. These variant callers often rely on external tools which align short reads to a reference genome to detect genetic variants. For example, the popular variant caller framework GATK [4] often used an external aligner known as BWA-SW [13] to align reads to reference genomes.

Although methods of aligning reads to genomes are diverse, they are essentially based on two important steps: finding seeds (which are exact matches between a substring of a read and substrings of the genome) and extending seeds into full alignments. Further, the extension of seeds into a full alignment often utilizes a technique based on the local pairwise sequence alignment [14]. Variant callers utilized alignments produced from aligners to call genetic variants that are different from the reference genome. In essence, each difference (substitution or gap) in a correct alignment results in a variant call (SNP or INDEL). Unfortunately, the basic algorithm of pairwise alignment does not account for multiple optimal alignments, each of which might result in different variant calls. From the theoretical point of view, each of the optimal alignments is equally likely to be the correct biological alignment. Thus, the choice of one optimal alignment over another is purely arbitrary.

In this paper, we demonstrate that many popular aligners can be divided into two groups. The first group of aligners produce alignments that would result in INDEL calls that agree with those reported in existing variant profiles, such as the resources curated by the 1000 Genomes Project. The second group of aligners produces alignments and INDEL calls that *disagree* with those reported in existing variant profiles. This finding implies that thousands of INDELs that have been reported in public resources were constructed based on algorithmic bias of alignment strategies. This source of bias adds to the list of biases in variant calling caused by sequencing technologies or coverage [15, 16]. It presents a problem for researchers who presume existing resources of human genetic variants as a gold standard for studying genetic variants.

## Methods

Methods that determine genetic variants from NGS data by and large rely on computational methods that align short reads to reference genomes and detect differences between them. The task of aligning short reads to genomes consists of two separate steps: (1) mapping reads to correct chromosomal locations and (2) aligning reads correctly to those chromosomal locations. A read can be correctly mapped and incorrectly aligned. Misalignment at a correct chromosomal location can affect the determination of insertion-deletion variants (INDEL). An INDEL is represented in the form *x*_1_|*x*_2_|⋯|*x*_*k*_, which means that at that location the string *x*_1_ appears in the reference genome, and any of *x*_1_,*x*_2_⋯*x*_*k*_ can appear in another genome at that location.

To see how a read can be correctly mapped and incorrectly aligned, consider an example, in which the read TCAGG is correctly mapped to the genome at location *p*, and that the substring starting at this location of length 8 is TCACACAG. Depending on the model of alignment, there are two or three different *optimal* alignments:



The first alignment results in 2 INDEL calls: TCA |T at location *p* and ACA |A at location *p*+4. The second alignment results in an INDEL call ACACA |A at location *p*+2. And the third alignment results in an INDEL call TCACA |T at location *p*.

In an alignment model where gap extensions and openings are equally penalized, these three alignments are all optimal because the gaps in each alignment equate to a deletion of 4 bases. In a model such as the *affine gap* model, in which a gap opening is penalized more than a gap extension, however, there are only two optimal alignments (the second and third) because the first alignment would be penalized more than the other two. So, even in the more sophisticated affine gap model, there can be multiple optimal alignments, resulting in different INDEL calls. And if an aligner picks one of these based on some algorithmic bias, this bias will end up in a biased calling of INDEL.

The goal of this work is to examine known INDEL locations and determine if those locations permit multiple optimal alignments. Further, for INDEL locations that permit multiple optimal alignments, we aim to examine the possibility that they were constructed in a biased manner based on biased alignments of many popular short-read aligners.

### Pairwise alignment

The mechanism by which aligners can create a biased alignment can be seen more easily by an examination of the basic pairwise alignment algorithm [14]. Although different alignment methods have different ways to speed up the mapping of reads to genomes, e.g. using an FM index or a hash table, the alignment itself is essentially the same formulation of optimal pairwise alignment, based on dynamic programming.

In a simple alignment model with no penalty for gap opening, an optimal alignment between *x*=*x*_1_⋯*x*_*n*_ and *y*=*y*_1_⋯*y*_*m*_ is found by constructing a matrix *M*, in which *M*[*i,j*] is the score of an optimal alignment between *x*_1_⋯*x*_*i*_ and *y*_1_⋯*y*_*j*_, for 1≤*i*≤*n* and 1≤*j*≤*m*. With *M*[*i*,0]=*i* and *M*[0,*j*]=*j*, the matrix *M* is constructed based on the following relation:

1 $$ M[i,j] = \max\left\{\begin{array}{cc} M[i-1,j-1] +~ match~(x_{i},y_{j})\\ M[i-1,j] +~ \epsilon \\ M[i,j-1] +~ \epsilon \\ \end{array}\right.  $$

where *m* *a* *t* *c* *h*(*x*_*i*_,*y*_*j*_) is the cost of substituting *x*_*i*_ for *y*_*j*_ and *ε* is the cost of deleting *x*_*i*_ or inserting *y*_*j*_.

In the affine gap model, finding an optimal alignment between *x* and *y* depends on the computation of three matrices *M,X*, and *Y*. Here, *M*[*i,j*] is the score of an optimal alignment between *x*_1_⋯*x*_*i*_ and *y*_1_⋯*y*_*j*_, where *x*_*i*_ is aligned with *y*_*j*_. *X*[*i,j*] is the score of an optimal alignment in which *x*_*i*_ aligns with a gap. And, *Y*[*i,j*] is the score of an optimal alignment in which *y*_*j*_ aligns with a gap. The computation of the three matrices can be done based on the following relations:

2 $$ M[i,j] = \max\left\{\begin{array}{ll} M[i-1,j-1] + ~match~(x_{i},y_{j})\\ X[i,j] \\ Y[i,j] \\ \end{array}\right.  $$

3 $$ X[i,j] = \max\left\{\begin{array}{ll} M[i-1,j] +~ (\epsilon + \rho) \\ X[i-1,j] +~ \epsilon \\ \end{array}\right.  $$

4 $$ Y[i,j] = \max\left\{\begin{array}{ll} M[i,j-1] +~ (\epsilon + \rho) \\ Y[i,j-1] +~ \epsilon \\ \end{array}\right.  $$

where *ε* is the cost of inserting or deleting a base, and *ρ* is the cost of inserting or deleting the first base (i.e. the penalty for gap opening).

### Constructing all optimal alignments

In Eqs. 1–4, when there exist more than one ways to achieve a maximal value, the choice adopted by an alignment algorithm will be arbitrary. Further, each arbitrary choice of maximal value of each step will lead to a specific optimal alignment. Thus, given the existence of more than one maximal cases to choose from in Eqs. 1–4, there will necessarily be multiple optimal alignments, which all have the same alignment scores despite being slightly different from one another.

To construct all optimal alignments under the non-affine gap model after the matrix *M* is filled, one starts from the entry with the highest cost and retraces all steps at which optimal decisions (as specified in Eq. 1) are made. The following procedure constructs all optimal alignments in the non-affine model, after the matrix *M* is computed: 1: Find (*i,j*) such that *M*[*i,j*] is maximum. 2: **return** *T* *r* *a* *c* *e*(*M,i,j*)

As described in Algorithm 1, the call *T* *r* *a* *c* *e*(*i,j*) returns all optimal alignments ending at *x*_*i*_ and *y*_*j*_. Trace is done by identifying whether each of the three conditions in Eq. 1 is optimal. If the condition is optimal, Trace is called recursive to obtain all optimal alignments starting at that entry. By induction, the three recursive calls return all possible optimal alignments just before *x*_*i*_ and *y*_*j*_. Then, the algorithm correctly returns the union of all of the optimal alignments ending at *x*_*i*_ and *y*_*j*_.



To construct all optimal alignments under the affine gap model, the process is similar. After the matrices *M,X*, and *Y* are filled, one starts from the entry of *M* with maximum value and retraces all the steps at which optimal decisions (as specified in Eqs. 2, 3, 4) are made. The only technical difference is that we need to specify the appropriate matrix (either *M*, *X*, or *Y*) in each recursive call.

## Experimental design

We hypothesize that the usage of an aligner to detect variants will result in incorporating the aligner’s bias into the construction of a variant profile. Specifically, this bias will exhibit itself at INDEL locations that have multiple optimal alignments. In our analysis the reference human genome, obtained from NCBI, build GRCh37, and the known variant profile obtained from the Integrated Variant Set release from the 1000 Genomes Project Consortium, we found that among 1,442,639 INDEL locations, 6,685 of them had multiple optimal alignments.

To demonstrate that many of these INDELs were created based on the bias of some alignment algorithms, we set out to reverse engineer the process of determining these INDELs based on various alignment algorithms. In the reverse engineering process, we create a set of reads $\mathcal {R}$ that bear alternative alleles from INDEL locations with more than one optimal pairwise alignments and use each aligner to align these reads to the reference genome. The alignment of each read in $\mathcal {R}$ to the correct INDEL location gives rise to a variant call. By recording the number of variant calls that agree with the known variant profile, we can compare the aligners’ degrees of agreement with known variant profiles and detect aligners’ bias, if there is any. Specifically, the process works as follows:

1. Suppose that the INDEL location *i* has two known alleles: *A* and *ACGA*, where *A* is in the reference genome, and *ACGA* is an alternative allele.
2. Suppose the reference genome *g* is represented as *xAy* (*g*_*i*_ is *A*).
3. Let *u* be a suffix of *x*, and *v* be a prefix of *y*. (Both presumably have length *k*). In other words, *u* and *v* are *k*-substrings of the genome that are on the left and the right of the allele *A*.
4. We will create a string *r*=*u* *A* *C* *G* *A* *v*. The string *r* is presumed to be the substring of another genome that differs from the reference genome at the exact location *i* with allele *ACGA*. We varied the length of *u* and *v* between 25 and 50. Thus, the length of the read *r* is around 50 to 100. (The actual length is equal to the length of *u* or *v* plus the length of the INDEL allele at location *i*).
5. Now if we align *r* to the reference genome, and if *r* is correctly mapped to location *i*, then two possible optimal alignments can be observed:

6. Of these two optimal alignments, the one on the left resulted in the variant *A*|*A* *C* *G* *A*, which agrees with the known profile. The other alignment resulted in a variant call at location *i*−1 that is different from the known profile. In general, there can be many optimal alignments but only one of them results in a variant call that agrees with the known variant profile.
7. If there are multiple alternative INDEL alleles at location *i*, each string *r* is created for each alternative allele.
8. Let $\mathcal {R}$ be the set of strings *r*’s that are constructed as we have described. For each INDEL location with more than one optimal pairwise alignments, there are exactly 10 reads with length between roughly 50 to 100, as described above, yielding a 10x coverage at those INDEL locations. To test whether the existing variant profile consists of INDELs that might have been constructed based on a bias alignment method, we employed several popular short-read aligners to all strings in $\mathcal {R}$.

## Result

To map and align reads in $\mathcal {R}$ to the reference genome, we considered several popular aligners: Bowtie2 [17], BWA-SW [13], CUSHAW2 [18], Smalt [19], SRmapper [20], SHRiMP2 [21], RazerS [22], GASSST [23], SeqAlto [24], Masai [25], and Soap2 [26]. Most aligners employed a seed-and-extend strategy, which first finds exact matches (seeds) between reads and the genome, and then extend such seeds to full alignments between reads and the genome. While these aligners adopt a wide range of algorithmic techniques in building indexes to facilitate efficient seed finding, the extension phase of their methods is based on the basic local alignment strategy, which is described in Introduction. We eliminated four aligners SeqAlto, Masai, Soap2, and SRmapper, due to their inability to map reads in $\mathcal {R}$ to their correct positions. Possible reasons include: (1) reads in $\mathcal {R}$ are relatively short and aligners might have been designed to work effectively with long reads, and (2) these reads might have been mapped to multiple chromosomal locations, and these aligners might have decided not to map any of them due to such confusion. For BWA, we used the BWA MEM version that is designed to work with both short and long reads.

### Analysis of INDELs with multiple optimal alignments

The set of reads $\mathcal {R}$ surrounding known INDEL locations were aligned by all aligners to the reference genome. For each aligner, we recorded the percentage of reads in $\mathcal {R}$ that the aligner was able to map to their correct locations. By design, each read covers a specific INDEL. A read is mapped correctly if it overlaps with the INDEL location that it is supposed to covers. Given that a read is mapped correctly, the alignment between the read and the genomic region gives rise to a unique variant call at that INDEL location. If there are more than one optimal pairwise alignments, the choice of which optimal alignment depends on the specifics of each alignment algorithm. As a result, the resulting variant call may or may not be the same with the reported variant profile that was created based on a different alignment algorithm.

As shown in Table 1, most aligners were able to map most reads in $\mathcal {R}$ to their correct INDEL locations with mapping percentages range from 88 to 97 %. Mapping a read to its correct INDEL location means that the read is mapped to a chromosomal location that overlaps the INDEL that the read was designed to cover. A correct mapping of a read does not mean that the alignment of the read to this location will yield a variant call that agrees with (or matches) the known variant profile. When there are multiple optimal alignments between a read and genomic fragment, each optimal alignment results in a different INDEL call. An alignment agrees with the existing information, if it produces an INDEL that is the same as the existing known INDEL. Table 1 reveals that these aligners can be divided into 3 groups:

1. Aligners whose correctly mapped reads (to INDEL locations with multiple optimal alignments) are aligned in high agreement with the known variant profile, about 99 % in agreement. These aligners include Bowtie2, BWA (MEM version), and SHRiMP2;
2. Aligners whose correctly mapped reads are aligned in moderate agreement with the known variant profile (between 70–75 %). These include RazerS and CUSHAW2; and
3. Aligners whose correctly mapped reads are aligned in high disagreement with the known variant profiles (less than 10 %). These include GASSST and Smalt.

**Table 1:** Percentage of correct mapping, actual and expected alignment by aligners

Aligners	Correct mapping %	Actual agreement %	Expected agreement %	*p*-value
Bowtie2	96	99	30	0.0000546
BWA	93	99	30	0.0000550
SHRiMP2	97	99	31	0.0001491
RazerS	88	75	31	0.0001631
CUSHAW2	97	70	31	0.0000562
GASSST	91	8	17	0.0015892
Smalt	96	5	31	0.0003852

To analyze if there exists alignment bias in reported variant profiles, we compare an aligner’s degree of agreement with reported variant profiles to the expected agreement if the algorithmic choice happens by chance. Suppose that at INDEL location *i*, there are *n*_*i*_ optimal pairwise alignments (under the affine-gap model), then the probability *p*_*i*_ that an aligner produces an alignment that yields a call in agreement with the known variant profile is ${1 \over n_{i}}$. The expected number of agreed calls is also ${1 \over n_{i}}$. Summing over all events, we find that the expected number of instances that agree with the known variant profile is $\sum _{i=1}^{N} {1 \over n_{i}}$, where *N* is the number of INDEL locations with multiple pairwise alignments that the aligner can map correctly reads in $\mathcal {R}$ to.

The last column of Table 1 shows the expected percentage of agreement by each aligner $\left ({1 \over N} \sum _{i=1}^{N} {1 \over n_{i}}\right)$. We can see that across all aligners, there is a significant different between the expected percentage of agreement and the actual agreement. For example, with Bowtie2, the expected percentage of alignment is 30 % compared to the actual percentage of agreement, which is 99 %. This vast difference between the expected and actual degree of agreement suggests that variant calls at these INDEL locations were obtained by alignment algorithms that were very similar to those aligners in the first groups (Bowtie2, BWA, SHRiMP2) whose actual percentage of agreement is more than 3 times the expected percentage of agreement. To compute the likelihood of this difference, we calculated the probability that the difference between the actual agreement and expected agreement (as happened by chance) is as much as or even more extreme than what we observed. This p-value can be bounded by the Chebyshev-Cantelli’s inequality, $P(X-\mu < \lambda) < {\sigma ^{2} \over \sigma ^{2} + \lambda ^{2}}$, where *λ* is the observed difference between actual and expected agreement, *μ* and *σ* are the expected agreement and its variance. As described above, $\mu = \sum _{i=1}^{N} {1 \over n_{i}}$. Further, $\sigma ^{2} = \sum _{i=1}^{N} {1 \over n_{i}}\left (1- {1\over n_{i}}\right)$. The very small p-values shown in the last column of Table 1 suggest that the difference in actual and expected agreement is extremely unlikely caused by chance.

### Characterization of INDEL complexity

The existence of multiple optimal alignments giving raise to different INDEL calls is an inherent problem. We have demonstrated that in many cases there are more than one theoretically optimal alignment, each of which has the same chance of being biologically correct. It is important to note that there is no correct optimal alignment among all possible optimal alignments: they are all optimal and thus equal probability of being the correct alignment. In other words, it does not matter which optimal alignment an aligner chooses and a variant caller utilizes the aligner’s result, there must be inevitably some bias. The only way to cope with this is for an aligner to report all optimal alignments and for a variant caller to derive all *alternative possibilities* of INDELs from these optimal alignments. This is tedious and not being done in practice. Existing variant profiles do not report alternative possibilities of INDELs; they only report one.

**Fig. 1:**
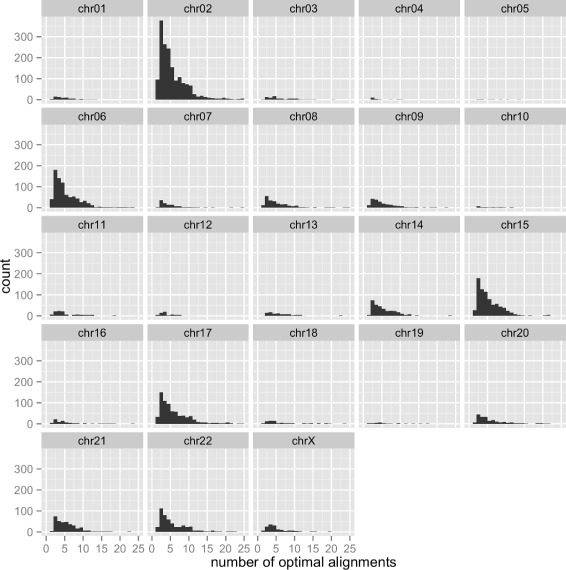
Distribution of INDEL complexity across human chromosomes

**Fig. 2:**
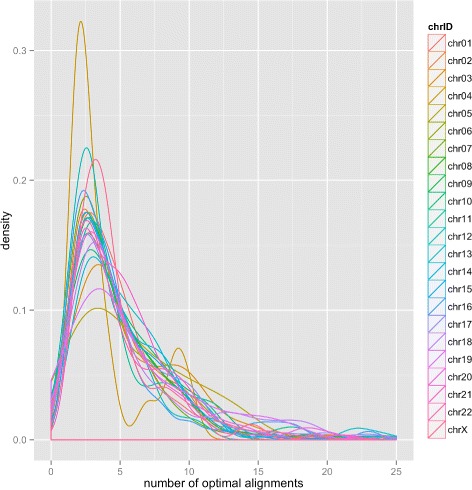
Density of INDEL complexity across human chromosomes

Thus, it is useful to examine known INDEL locations and characterize the extent to which they are affected by multiple optimal alignments. We define the *complexity of each INDEL location* as the number of optimal alignments that can be had when reads bearing alternative alleles are aligned (under the affine-gap model) to the reference genome at this location. Figure 1 shows the distribution of INDEL complexity across human chromosomes. We observed that chromosome Y has no INDEL with multiple optimal alignments. Further, a closer examination of the density of INDEL complexity on all chromosomes, as shown in Fig. 2, suggests that these distributions are very similar, with the peak occurs at around 3. A majority of these INDELs have 3 multiple optimal alignments. Additionally, chromosomes 2, 6, 15, and 16 stood out with the most number of INDEL locations with multiple optimal alignments. Larger chromosomes do not necessarily have more complex INDELs. For example, compared to the others, chromosome 1 has fewer INDELs with multiple optimal alignments.

## Discussions and conclusions

The accuracy of calling variants can be improved by increasing coverage (i.e. using more reads) and realigning reads that overlap INDEL locations. But we argue that neither increasing coverage nor realigning reads around INDELs can help resolve the problem caused by multiple optimal alignments. Increasing reads can reduce the damaging effect of sequencing errors, which occur independently across reads. While realigning reads around an INDEL as described by Li [27] can achieve a better multiple alignment of reads aligned to the INDEL, the multiple alignment is still biased as it is based on one of the optimal pairwise alignments. For instance, recall the example given earlier, in which the read TCAGG is correctly mapped to the genome and is aligned to the genomic sequence TCACACAG. As we showed earlier, there are multiple optimal pairwise alignments. Let us supposed that many reads are aligned to this region. It is possible (due to different chromosomal positions), the alignments of some reads might look like the first alignment in this example; the alignments of some other reads might look like the second alignment; and the alignments of the rest might look like the third alignment. The goal of realigning reads [27], which were pairwise aligned, is to obtain a consistent multiple alignment of reads. The result of such realignment would be an adoption of the same alignment for all reads aligned to this region to obtain a high quality call. But the adopted multiple alignment is still based on one of the three optimal pairwise alignments. As such, the realignment of reads still produces biased results.

We have demonstrated that the current INDEL profile constructed and curated by the 1000 Genome Project exhibits a bias at certain INDEL locations. These locations can be identified by counting the number of optimal alignments between reads containing alternative alleles to the reference genome at those locations. The bias is essentially an effect of either short-read aligners or variant callers themselves having to choose one out of many equally theoretically optimal alignments. There is no obvious way to “standardize" this phenomenon by designating one optimal alignments as the “canonical" one. As such, it seems the only way to deal with this is reporting all optimal alignments and consequently reporting all alternative INDEL calls as the result of those alignments.

If this phenomenon is not addressed, there can be potential serious problems relating to the analysis and study of INDEL. For example, certain alignment techniques will result in wrong calls at those INDEL locations. Case in point is Smalt, which was able to map 96 % of the reads, but very few of the alignments produced the “correct" INDEL calls (as specified by the existing INDEL information). At these location, Smalt was wrong simply because it chooses a different optimal alignment from the one based on which the INDEL was constructed.
